# Field-assessed functional exercise capacity and selected NCD-related health markers among students at Kuwait University: a cross-sectional study using the incremental shuttle walk test

**DOI:** 10.3389/fspor.2026.1852703

**Published:** 2026-08-12

**Authors:** Ahmad Salman

**Affiliations:** Department of Public Health Practice, College of Public Health, Kuwait University, Kuwait City, Kuwait

**Keywords:** body mass index, functional exercise capacity, gulf cooperation council, incremental shuttle walk test, Kuwait, non-communicable diseases, physical activity, university students

## Abstract

**Background:**

Functional exercise capacity, assessed as observed distance on the Incremental Shuttle Walk Test (ISWT), is a field-based indicator relevant to cardiometabolic health, yet it remains poorly characterised among university students in Gulf Cooperation Council (GCC) countries. This cross-sectional study characterised field-assessed functional exercise capacity and selected NCD-related health markers among students at Kuwait University.

**Methods:**

A pragmatic convenience sample of 389 Kuwait University students (76.3% female; mean age 20.2 ± 2.3 years) completed the ISWT between 28 July and 4 December 2025, alongside assessments of physical activity (IPAQ-SF), depressive symptoms (PHQ-9), anthropometric measurements, and resting clinical indicators. The primary outcome was observed ISWT distance (metres). Physical-activity volume was expressed as log₁₀-transformed total MET-min/week. Hierarchical multiple linear regression was used to identify independent correlates of observed ISWT distance; equation-derived percentage-predicted outcomes were examined only as exploratory supplementary analyses restricted to participants aged ≥21 years.

**Results:**

Mean observed ISWT distance was 439.1 ± 306.0 m (median 360 m, IQR 230–550 m). Males walked significantly further than females (596.7 ± 388.1 m vs. 390.3 ± 257.5 m, *p* < 0.001). In the fully adjusted primary model, male sex (*β* = 0.283, *p* < 0.001), lower body mass index (*β* = −0.162, *p* < 0.001), higher physical-activity volume (log₁₀ MET-min/week; *β* = 0.113, *p* = 0.023), and Kuwaiti nationality (*β* = −0.099, *p* = 0.040) were independently associated with observed ISWT distance, explaining 13.3% of variance (adjusted R2 = 0.120). Depressive symptoms were not independently associated with observed ISWT distance (*β* = −0.079, *p* = 0.104). An exploratory sex×physical-activity interaction was observed (*p* = 0.010), with the positive activity association evident among females but not the smaller male subgroup.

**Conclusion:**

Observed ISWT distance was independently associated with male sex, lower BMI, higher physical-activity volume, and nationality among students at Kuwait University. Although the single-university cross-sectional design precludes policy-level inference, these findings identify physical-activity volume and body composition as potentially modifiable correlates and support the development and evaluation of campus-based physical-activity and early NCD-prevention initiatives in Kuwaiti higher-education settings.

## 1 Introduction

Cardiorespiratory fitness (CRF) represents the integrated capacity of the cardiovascular and respiratory systems to deliver oxygen to working muscles during sustained physical activity, and serves as one of the strongest independent predictors of all-cause and cardiovascular mortality across the lifespan. An umbrella review of 26 systematic reviews encompassing over 20.9 million observations from 199 unique cohort studies demonstrated that high CRF is associated with a 53% reduction in all-cause mortality risk compared to low CRF (HR = 0.47; 95% CI 0.39–0.56), with a dose-response relationship whereby each one-metabolic equivalent increment in CRF is associated with an 11%–17% reduction in all-cause mortality risk (1). These findings are corroborated by an updated meta-analysis of 37 cohort studies involving 2,258,029 participants, which reported a pooled multivariable-adjusted relative risk of 0.55 (95% CI 0.50–0.61) for all-cause mortality comparing the highest to lowest CRF tertiles (2). At an individual level, a large cohort study of 122,007 adults undergoing exercise treadmill testing found an inverse, dose-dependent association between CRF and all-cause mortality with no observed upper limit of benefit, with the lowest-performing group demonstrating over five times the adjusted mortality risk of the highest performers (3). In light of this evidence, the American Heart Association has formally proposed CRF as a clinical vital sign, reflecting the integrated functional capacity of the cardiovascular, respiratory, and musculoskeletal systems and its potent prognostic significance beyond traditional risk factors (4). Of note, the combination of obesity and low CRF confers particularly elevated cardiovascular disease risk, whereas increasing CRF has been shown to markedly attenuate or even nullify the adverse cardiovascular effects associated with obesity (5).

Globally, physical inactivity and declining CRF have emerged as critical public health challenges, with the World Health Organization identifying physical inactivity as a leading modifiable risk factor for premature mortality and non-communicable disease (*N*CD) development. The Gulf Cooperation Council (GCC) region, including Kuwait, faces a particularly severe NCD burden, with relatively few physical activity intervention studies conducted despite the urgent need (6). Kuwait has a substantial burden of physical inactivity, obesity, and NCD mortality: physical inactivity rates reached 60% in males and 73% in females as of 2016, NCDs account for an estimated 72% of all deaths, and over one-third of adults are classified as obese — among the highest proportions globally (7). National time-series analyses confirm that cardiometabolic mortality remained persistently elevated in Kuwait between 2010 and 2022 despite health system expansion, suggesting that upstream behavioural risk factors continue to drive disease burden (8). Across the Eastern Mediterranean region, rising rates of obesity represent a growing public health emergency requiring urgent targeted action to reduce the associated NCD burden (9).

University students represent a strategically important population for early NCD risk identification and prevention. The transition to higher education is frequently characterised by declining structured physical activity, increased sedentary academic workloads, disrupted sleep patterns, and elevated psychosocial stress, collectively creating conditions that may compromise CRF during what should be peak physiological capacity. Systematic reviews confirm that physical activity interventions are effective in reducing symptoms of anxiety, depression, and perceived stress among university students, underscoring the bidirectional importance of physical and psychological health during this life stage (10). A scoping review of university students further demonstrates that sedentary behaviour, low physical activity, and academic stress are strongly interrelated, with higher levels of sitting time associated with poorer stress and mental health outcomes (11). Cross-national survey data from 23 countries indicate that physical inactivity affects a substantial proportion of university students, with prevalence estimates ranging widely by country, income level, and sex (12). CRF has been identified as a potential pathway linking physical activity and mental health among university students, suggesting that improving CRF may be one mechanism through which physical activity confers psychological benefits (13). These findings highlight the importance of understanding CRF not only as a cardiovascular risk marker but also as a modifiable correlate of psychological wellbeing in young adults.

The Incremental Shuttle Walk Test (ISWT), originally developed by Singh et al. (14) as an externally paced, progressive field test, provides a standardised and objective field-based measure of functional exercise capacity. The ISWT demonstrates valid and reliable performance characteristics in both clinical and healthy populations: studies in cardiac rehabilitation have confirmed its responsiveness to clinically meaningful change (15), and reference values derived from 242 healthy subjects across a broad age range confirm that sex, age, and BMI are the primary independent determinants of ISWT distance, together explaining 71% of its variance (16). These properties collectively make the ISWT practically suitable for population-based assessment without specialised laboratory equipment. Reference equations enabling individual performance to be expressed relative to age- and sex-adjusted predicted values have been established across diverse populations, including a validated equation derived from a healthy Asian adult cohort aged 21–80 years (R^2^ = 0.741) that was applied in the present study (17). Despite the ISWT's utility, to our knowledge, no published study has applied it to characterise functional exercise capacity or its independent correlates among university students in Kuwait or the broader GCC region, where objective field-based fitness benchmarks and multidimensional health-marker profiles remain undefined.

The present study aimed to characterise field-assessed functional exercise capacity, measured as observed ISWT distance, together with selected NCD-related health markers among students at Kuwait University, and to identify their independent sociodemographic, anthropometric, behavioural, and psychological correlates in this under-studied population.

## 2 Materials and methods

This was a cross-sectional study conducted at Kuwait University, Kuwait City, Kuwait. Data collection took place between 28 July 2025 and 4 December 2025 using a standardised face-to-face protocol administered on campus. The study was conducted in accordance with the Declaration of Helsinki. Ethical approval was obtained from the Health Sciences Centre Ethics Committee (HSC-EC), Kuwait University (Approval No. VDR/EC-2025-114; approved 26 May 2025). Written informed consent was obtained from all participants prior to enrolment.

Participants were undergraduate and postgraduate students enrolled at Kuwait University. A pragmatic convenience sampling approach was used: all eligible students who provided informed consent and completed the ISWT during the study period were included. Potential participants were recruited through posted notices, in-class announcements, and word-of-mouth referrals across colleges. Eligibility was determined using the Physical Activity Readiness Questionnaire Plus (PAR-Q+), which screened for contraindications to exercise testing. Students were eligible if they were aged 18 years or older, able to understand the English-language study procedures and questionnaire, and free from any condition that would preclude safe exercise testing. Exclusion criteria included any affirmative response to the PAR-Q+, acute musculoskeletal injury, pregnancy, or physician-advised restriction from physical activity. No exclusions were applied based on sex, nationality, body weight, or academic year. No formal *a priori* sample size calculation was performed. A total of 390 responses were received. One record was excluded before creation of the final analytic dataset. No additional records required exclusion after derivation and validation of the 389 retained records, which included two graduate students retained because no exclusion criteria were based on academic year.

Height was measured to the nearest 0.1 cm using a stadiometer with participants standing without footwear. Body weight was measured to the nearest 0.1 kg using a calibrated digital scale. Body mass index (BMI) was calculated as weight in kilograms divided by height in metres squared (kg/m^2^) and categorised according to World Health Organization criteria as underweight (<18.5 kg/m^2^), normal weight (18.5–24.9 kg/m^2^), overweight (25.0–29.9 kg/m^2^), and obese (≥30.0 kg/m^2^). Waist and hip circumferences were measured to the nearest 0.5 cm using a non-elastic tape measure following standardised landmarks, and waist-to-hip ratio (WHR) was calculated. Resting heart rate (HR) and peripheral oxygen saturation (SpO₂) were measured after five minutes of seated rest using a digital pulse oximeter. Resting systolic (SBP) and diastolic (DBP) blood pressure were measured using a calibrated digital sphygmomanometer. Pulse pressure was derived as SBP minus DBP.

Functional exercise capacity was assessed as observed distance on the Incremental Shuttle Walk Test (ISWT), originally developed by Singh et al. (1992) (14). Observed ISWT distance is interpreted as a field-assessed measure of functional exercise capacity and is not equated with direct measurement of maximal cardiorespiratory fitness or VO₂max. The test was conducted along a 10-metre flat course marked by two cones, using a standardised audio signal to dictate walking pace. Speed increased progressively each minute according to the standard protocol. The assessor recorded a single termination category for each participant from six predefined options: an assessor-recorded age-predicted maximum-heart-rate stop criterion (220−age), an assessor-recorded oxygen-saturation stop criterion (recorded in the data-collection form as SpO₂ < 80%–85%), inability to maintain the required pace (missed two consecutive shuttles), fatigue or breathlessness precluding continuation, clinical symptoms, or a rating of perceived exertion of 9–10 with inability to continue; an optional free-text “Other” field was also available. The total number of completed shuttles was recorded, and observed ISWT distance (metres) was calculated as the number of shuttles multiplied by 10. Termination reason was recorded as a single nominal variable. A modified Borg scale rating of perceived exertion (RPE) was recorded before and immediately after the test (scale 0–10) (18). Heart rate was recorded immediately post-test, and peripheral oxygen saturation was recorded after the test. Heart rate at one minute of passive recovery (HR_Recovery) was also recorded, and the HR recovery drop was calculated as post-exercise HR minus one-minute recovery HR. All participants completed PAR-Q + screening before testing, and pre-test heart rate, peripheral oxygen saturation, and systolic and diastolic blood pressure were recorded. ISWT testing was supervised by the study team in accordance with the study protocol. No serious adverse events were recorded during testing.

As an exploratory supplementary analysis restricted to participants aged ≥21 years (the derivation age range of the reference equation), ISWT performance was additionally expressed as a percentage of individually predicted distance and as the observed-minus-predicted difference. Predicted ISWT distance was calculated using the reference equation for healthy Asian adults aged 21–80 years developed by Azman et al. (2023) (17):

ISWT_pred_ = 651.4 × Height (m) + 89.7 × Sex (male = 1, female = 0) − 6.31 × Age (years) − 3.61 × Weight (kg) + 2.54 × ΔHR.

where *Δ*HR = post-exercise HR minus resting HR. Percentage of predicted ISWT was calculated as observed ISWT distance divided by predicted distance, multiplied by 100. Because the reference equation was derived in adults aged 21–80 years and was not validated in this population or in participants younger than 21 years, and because *Δ*HR is an effort-dependent term embedded in the equation, all equation-derived outcomes are reported only as exploratory supplementary analyses (Supplementary Table 1); no percentage-predicted classification categories are applied in the main analysis.

Habitual physical activity was assessed using the International Physical Activity Questionnaire—Short Form (IPAQ-SF) (19, 20), a self-administered questionnaire that captures frequency, duration, and intensity of vigorous-intensity, moderate-intensity, and walking activity performed during the preceding seven days, as well as weekday sitting time. Total physical activity was expressed in metabolic equivalent task minutes per week (MET-min/week), calculated using the standard IPAQ scoring protocol: vigorous activity  ×  8.0 METs, moderate activity×4.0 METs, and walking×3.3 METs (20). For descriptive purposes, participants were grouped by total MET-min/week into low (<600), moderate (600–2,999), and high (≥3,000) volume groups; because full IPAQ categorical scoring using activity-day and duration criteria could not be independently verified, this variable is treated as a descriptive study-defined volume grouping rather than a formal IPAQ category. In regression analyses, physical-activity volume was entered as the continuous log₁₀-transformed total MET-min/week [log₁₀(MET-min/week + 1)], which reduced skew and served as the primary physical-activity exposure.

Depressive symptoms were assessed using the Patient Health Questionnaire-9 (PHQ-9) (21), a validated nine-item self-report instrument yielding a total score from 0 to 27, with higher scores indicating greater symptom burden; PHQ-9 total score was entered as a continuous covariate in regression analyses. Psychological wellbeing was additionally assessed using the World Health Organisation Five-Item Wellbeing Index (WHO-5) (22), a brief self-report scale producing a percentage score from 0 to 100, with lower scores indicating poorer wellbeing; WHO-5 was retained as a descriptive and secondary correlate variable.

Sociodemographic data collected included age (years), sex (male/female), nationality (Kuwaiti/non-Kuwaiti), and academic year. Smoking status was recorded as never, former, occasional, or daily smoker, and vaping status was similarly classified. Dietary habits and sleep quality were collected via brief self-report items but are not primary exposures in the present analyses; these variables are reserved for subsequent papers in this programme of research.

A standardised data cleaning protocol was applied prior to analysis. A validity flag (CleanFlag) was assigned to each record; participants were excluded if any of the following criteria were met: pre-test SpO₂ > 100%, resting SBP <80 mmHg, waist circumference >150 cm, or ISWT distance >1,500 m, based on physiologically implausible values and predefined data quality thresholds. All derived variables (BMI, WHR, IPAQ MET scores, PHQ-9 total, WHO-5 raw and percentage scores) were independently recalculated from item-level data and verified against stored values. As noted above, one record was excluded before creation of the final analytic dataset; no additional records required exclusion after derivation and validation of the 389 retained records.

All statistical analyses were conducted using IBM SPSS Statistics version 31.0.1.0. Continuous variables are presented as means ± standard deviations (SD), with medians and interquartile ranges (IQR) reported for non-normally distributed variables. Categorical variables are presented as frequencies and percentages. Normality was assessed using the Shapiro–Wilk test and visual inspection of histograms and normal probability plots.

Differences between sexes in continuous variables were examined using independent samples *t*-tests for normally distributed variables, and Mann–Whitney U tests for non-normally distributed variables; all continuous variables were tested for normality prior to group comparisons. ISWT distance, percentage predicted ISWT, and IPAQ total MET-min/week were non-normally distributed (Shapiro–Wilk *p* < 0.001) and were therefore compared between sexes using Mann–Whitney U tests, with results additionally consistent with parametric testing (data not shown). Differences in categorical variables by sex were examined using chi-square tests. Spearman rank correlations were used to examine bivariate associations between ISWT distance and physical activity, depressive symptoms, and anthropometric variables.

A hierarchical multiple linear regression model was constructed to identify independent correlates of observed ISWT distance (the primary outcome). Predictors were entered in three sequential blocks on theoretical grounds: Block 1 entered sociodemographic and anthropometric variables (sex, age, BMI, nationality); Block 2 added depressive symptoms (PHQ-9 total score), a pre-specified predictor; and Block 3 added physical-activity volume (log₁₀ MET-min/week). This sequencing was specified *a priori* to isolate the independent contribution of behavioural and psychological variables after demographic and body-composition factors were accounted for. Variables were coded as sex (male = 1, female = 0), Kuwaiti nationality (Kuwaiti = 1, non-Kuwaiti = 0), with PHQ-9 and log₁₀ MET-min/week entered as continuous. An exploratory model additionally tested sex×PHQ-9, sex×BMI, and sex×physical-activity interaction terms using mean-centred predictors, and sex-stratified models were examined (Supplementary Table 2).

Equation-derived outcomes (percentage of predicted ISWT distance and observed-minus-predicted difference) were examined only as exploratory supplementary analyses restricted to participants aged ≥21 years (Supplementary Table 1). Sex, age, height, and weight were not entered as covariates in equation-derived models because they are components of the prediction equation used to construct the outcome; including them would create mathematical coupling and complicate interpretation.

Missing data were handled using listwise deletion. All 389 participants had complete data for the primary observed-ISWT model. Reduced sample sizes in age-restricted and sex-stratified analyses reflected prespecified subgroup restrictions rather than missing data. Model fit was assessed using R^2^ and adjusted R^2^, with change statistics (*Δ*R^2^, *Δ*F) reported for each block. Regression coefficients are presented as unstandardised (B) and standardised (*β*) values with 95% confidence intervals. Multicollinearity was assessed using tolerance and the variance inflation factor (VIF). Visual inspection of the residual histogram, normal P–P plot, and residual-vs.-fitted plot was used to check linear-model assumptions (Supplementary Figure 1); these plots showed approximately normal residuals and no systematic heteroscedasticity. No case was identified by casewise diagnostics as having an absolute standardised residual exceeding 3.0. To evaluate robustness, the following analyses were conducted: BCa bootstrap replication of the primary model (5,000 samples); an age-restricted analysis (age 18–24 years); and, as exploratory supplementary analyses restricted to participants aged ≥21 years, regression of observed ISWT distance on predicted distance, the observed-minus-predicted difference, and percentage of predicted ISWT distance. Statistical significance was set at *p* < 0.05 (two-tailed). All analyses were conducted using IBM SPSS Statistics version 31.0.1.0.

## 3 Results

### 3.1 Sample characteristics

A total of 389 students were included in the final analytic sample (297 female, 76.3%; 92 male, 23.7%). The mean age was 20.2 ± 2.3 years (range 18–42); the age distribution was positively skewed (Shapiro–Wilk W = 0.717, *p* < 0.001), reflecting the predominantly undergraduate composition of the sample, with 81.5% of participants enrolled in their first three years of study and only 0.5% graduate students. The majority were Kuwaiti nationals (80.2%). Current smoking was reported by 6.4% of participants and current vaping by 6.9%.

Mean body mass index (BMI) was 25.4 ± 5.5 kg/m^2^. Based on World Health Organization criteria, 8.2% of participants were classified as underweight, 42.4% as normal weight, 29.8% as overweight, and 19.5% as obese. Mean waist circumference was 75.8 ± 16.1 cm and mean waist-to-hip ratio was 0.77 ± 0.08. Resting systolic blood pressure was 118.2 ± 12.7 mmHg, resting diastolic blood pressure was 79.2 ± 10.6 mmHg, and resting heart rate was 85.4 ± 12.6 bpm. Resting peripheral oxygen saturation was 98.5 ± 0.9%. Mean PHQ-9 total score was 8.3 ± 4.6 (range 0–24) and mean WHO-5 wellbeing percentage score was 56.1 ± 16.2 (range 12–100). Detailed sample characteristics are presented in Table 1.

**Table 1:** Sample characteristics by sex (*N* = 389).

Variable	Overall (*N* = 389)	Female (*n* = 297)	Male (*n* = 92)	*p*-value
Sociodemographic characteristics				
Age (years), median (IQR)ᵃ	20 (19–21)	20 (19–21)	21 (19–22)	<.001
Kuwaiti nationality, *n* (%)	312 (80.2%)	238 (80.1%)	74 (80.4%)	.942
Academic year, *n* (%)	—	—		.692
1st year	91 (23.4%)	73 (24.6%)	18 (19.6%)	—
2nd year	101 (26.0%)	74 (24.9%)	27 (29.3%)	—
3rd year	125 (32.1%)	94 (31.6%)	31 (33.7%)	—
4th year or above	72 (18.5%)	56 (18.9%)	16 (17.4%)	—
Current smoker, *n* (%)	25 (6.4%)	2 (0.7%)	23 (25.0%)	<.001
Current vaper, *n* (%)	27 (6.9%)	5 (1.7%)	22 (23.9%)	<.001
Anthropometric and clinical measurements				
Height (cm), mean ± SD	161.7 ± 7.9	158.4 ± 5.3	172.2 ± 5.4	<.001
Weight (kg), mean ± SD	66.9 ± 17.2	62.5 ± 13.3	81.0 ± 20.6	<.001
BMI (kg/m^2^), mean ± SD	25.4 ± 5.5	24.8 ± 5.0	27.3 ± 6.5	<.001
BMI category, *n* (%)	—	—		.003ᵇ
Underweight (<18.5)	32 (8.2%)	25 (8.4%)	7 (7.6%)	—
Normal weight (18.5–24.9)	165 (42.4%)	138 (46.5%)	27 (29.3%)	—
Overweight (25.0–29.9)	116 (29.8%)	87 (29.3%)	29 (31.5%)	—
Obese (≥30.0)	76 (19.5%)	47 (15.8%)	29 (31.5%)	—
Waist circumference (cm), mean ± SD	75.8 ± 16.1	70.3 ± 11.4	93.6 ± 16.3	<0.001
Hip circumference (cm), mean ± SD	97.4 ± 14.2	94.4 ± 12.5	106.9 ± 15.2	<0.001
Waist-to-hip ratio, mean ± SD	0.77 ± 0.08	0.74 ± 0.06	0.87 ± 0.05	<0.001
Resting SBP (mmHg), mean ± SD	118.2 ± 12.7	116.2 ± 11.6	124.3 ± 14.2	<0.001
Resting DBP (mmHg), mean ± SD	79.2 ± 10.6	78.6 ± 10.1	81.0 ± 11.8	0.055
Resting HR (bpm), mean ± SD	85.4 ± 12.6	85.9 ± 11.6	84.1 ± 15.5	0.231
Resting SpO₂ (%), mean ± SD	98.5 ± 0.9	98.5 ± 1.0	98.5 ± 0.9	0.889
Physical activity (IPAQ-SF)				
Total MET-min/week, median (IQR)^a^	540 (198–1,548)	450 (148–1,070)	1,576 (588–3,889)	<0.001
Physical-activity volume group, *n* (%)	—	—		<0.001
Low (<600 MET-min/week)	205 (52.7%)	180 (60.6%)	25 (27.2%)	—
Moderate (600–2,999)	128 (32.9%)	94 (31.6%)	34 (37.0%)	—
High (≥3,000)	56 (14.4%)	23 (7.7%)	33 (35.9%)	—
Psychological variables				
PHQ-9 total score, mean ± SD	8.3 ± 4.6	8.8 ± 4.8	6.9 ± 3.7	<0.001
WHO-5 wellbeing score, mean ± SD	56.1 ± 16.2	55.1 ± 16.2	59.4 ± 15.9	—ᶜ

BMI, body mass index; DBP, diastolic blood pressure; HR, heart rate; IQR, interquartile range; IPAQ-SF, International Physical Activity Questionnaire—Short Form; MET, metabolic equivalent task; PHQ-9, Patient Health Questionnaire-9; SBP, systolic blood pressure; SD, standard deviation; SpO₂, peripheral oxygen saturation; WHO-5, World Health Organisation Five-Item Wellbeing Index. *p*-values derived from independent samples t-tests for normally distributed continuous variables, Mann–Whitney U tests for non-normally distributed continuous variables, and chi-square tests for categorical variables.

a Mann–Whitney U test; Shapiro–Wilk *p* < 0.001. ^b^BMI category chi-square: χ² = 13.883, df = 3, *p* = 0.003. ^c^WHO-5 sex comparison not performed; retained as a descriptive variable only. Academic-year chi-square is for the displayed four-category grouping (fourth year, fifth year, and graduate students collapsed into “4th year or above”): *χ*^2^ = 1.459, df = 3, *p* = 0.692. Physical-activity volume group: *χ*^2^ = 54.096, df = 2, *p* < 0.001.

### 3.2 Physical activity levels

Total physical activity was highly right-skewed (Shapiro–Wilk *p* < 0.001; median 540 MET-min/week, IQR 198–1,548). Over half of participants had low study-defined total-activity volume (<600 MET-min/week; 52.7%), while 32.9% had moderate and 14.4% had high total-activity volume. Males recorded significantly higher total-activity volume than females (Mann–Whitney U = 7,402, Z = −6.65, *p* < 0.001), with a markedly higher proportion of males in the high total-activity-volume group (35.9% vs. 7.7%) and a lower proportion in the low total-activity-volume group (27.2% vs. 60.6%).

### 3.3 Observed ISWT performance and termination reasons

**Figure 1:**
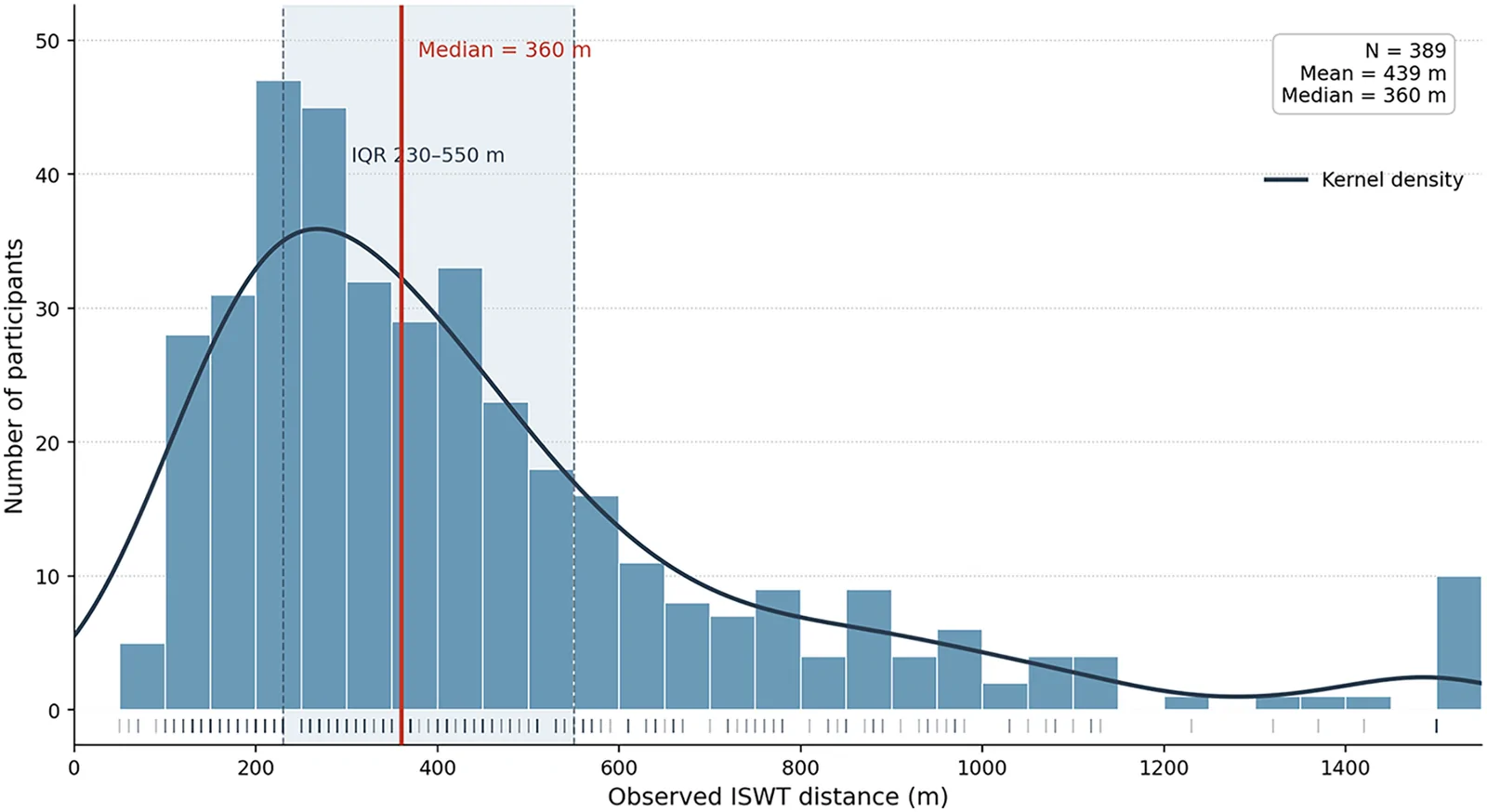
Distribution of observed incremental shuttle walk test (ISWT) distance in the study sample (*N* = 389). The histogram displays the distribution of observed ISWT distance in metres. The distribution was positively skewed (Shapiro–Wilk W = 0.840, *p* < 0.001), with a mean of 439.1 ± 306.0 m and a median of 360 m (IQR 230–550 m).

Mean observed ISWT distance was 439.1 ± 306.0 m (median 360 m, IQR 230–550 m), with values exhibiting marked positive skew (Shapiro–Wilk W = 0.840, *p* < 0.001; Figure 1). Males walked significantly greater distances than females (Mann–Whitney U = 8,876, Z = −5.08, *p* < 0.001; mean 596.7 ± 388.1 m vs. 390.3 ± 257.5 m). Termination status was recorded using six predefined form options and an optional free-text “Other” field. Recorded entries were inability to maintain pace or missed two shuttles (*n* = 115, 29.6%), combined clinical symptoms (*n* = 115, 29.6%), fatigue or breathlessness (*n* = 75, 19.3%), the assessor-recorded age-predicted maximum-heart-rate stop criterion (*n* = 63, 16.2%), the assessor-recorded oxygen-saturation stop criterion (*n* = 14, 3.6%), RPE 9–10 with inability to continue (*n* = 1, 0.3%), and six free-text “Other” entries indicating test completion (*n* = 6, 1.5%). Clinical symptoms were recorded as one pre-existing combined response option (chest pain, dizziness, cramps, or severe dyspnoea) and could not be disaggregated retrospectively. Sex-specific stop-reason frequencies are reported descriptively in Supplementary Table 3; no inferential comparison was interpreted because of sparse category counts.

Pre-test Borg RPE was 0 (nothing at all) for 94.9% of participants, confirming participants were at rest prior to testing. Post-test Borg RPE reflected a broad range of perceived exertion, with the majority of participants rating exertion at level 6 or above (65.8%); the modal response was level 7 (very hard; 22.6%). Mean post-exercise heart rate was 128.6 ± 25.3 bpm, and mean heart rate at one minute of passive recovery was 103.9 ± 21.0 bpm, yielding a mean heart rate recovery drop of 24.7 ± 14.0 bpm. Recorded post-test SpO₂ was 98.2 ± 1.3%; no recorded post-test value was below 80%.

### 3.4 Exploratory equation-derived analyses (age ≥21 years)

Among the 133 participants aged ≥21 years (the derivation age range of the reference equation), equation-derived percentage-predicted and observed-minus-predicted difference outcomes were examined as exploratory supplementary analyses (Supplementary Table 1). These equation-derived outcomes are not interpreted as clinical or population-specific fitness classifications and are not applied in the main analysis, because the reference equation was not validated in this population or in participants younger than 21 years and incorporates an effort-dependent *Δ*HR term. Table 2 presents a comparison of ISWT performance and related variables by sex.

**Table 2:** ISWT performance and exercise response by sex (*N* = 389).

Variable	Overall (*N* = 389)	Female (*n* = 297)	Male (*n* = 92)	Test statistic	*p*-value
ISWT performance					
Shuttles completed, mean ± SD (median, IQR)	43.9 ± 30.6 (36, 23–55)	39.0 ± 25.8	59.7 ± 38.8	U = 8,876; Z = −5.08ᵃ	<0.001
ISWT distance (m), mean ± SD (median, IQR)	439.1 ± 306.0 (360, 230–550)	390.3 ± 257.5	596.7 ± 388.1	U = 8,876; Z = −5.08^a^	<0.001
ISWT protocol — exercise response					
Post-exercise HR (bpm), mean ± SD	128.6 ± 25.3	129.2 ± 24.5	126.6 ± 27.8	t(387) = 0.88	0.384
Post-exercise SpO₂ (%), mean ± SD	98.2 ± 1.3	98.2 ± 1.3	98.1 ± 1.3	t(387) = 0.14	0.883
1-min recovery HR (bpm), mean ± SD	103.9 ± 21.0	103.5 ± 20.7	105.2 ± 21.7	t(387) = −0.65	0.514
HR recovery drop (bpm), mean ± SD	24.7 ± 14.0	25.7 ± 14.2	21.4 ± 12.8	t(387) = 2.58	0.010
Borg RPE pre-test, *n* (%) score 0	369 (94.9%)	—	—	—	—
Borg RPE post-test, median (IQR)	7 (5–8)	—	—	—	—

HR, heart rate; IQR, interquartile range; ISWT, Incremental Shuttle Walk Test; RPE, rating of perceived exertion; SD, standard deviation; SpO₂, peripheral oxygen saturation; U, Mann–Whitney U statistic.

a Mann–Whitney U test (primary test for all ISWT variables; Shapiro–Wilk *p* < 0.001). Because ISWT shuttles and distance are a linear transformation of one another (distance=shuttles×10), rank order is identical and U and Z statistics are equivalent. Results consistent with parametric analysis (data not shown). t-tests used for post-exercise HR, SpO₂, and HR recovery drop (normally distributed or adequate approximation at *n* = 389).

### 3.5 Bivariate associations with ISWT distance

Spearman rank correlations showed that observed ISWT distance was positively associated with total physical activity (*ρ* = 0.210, *p* < 0.001) and weakly negatively associated with PHQ-9 total score (*ρ* = −0.122, *p* = 0.016). WHO-5 wellbeing score was not significantly correlated with ISWT distance (*ρ* = 0.070, *p* = 0.170).

### 3.6 Hierarchical regression — observed ISWT distance

The hierarchical regression model for observed ISWT distance is summarised in Table 3. Block 1 (sex, age, BMI, nationality) explained 11.6% of variance (R^2^ = 0.116, adjusted R^2^ = 0.107, *p* < 0.001). The addition of PHQ-9 in Block 2 did not significantly increase explained variance (*Δ*R^2^ = 0.006, *p* = 0.115). The addition of physical-activity volume (log₁₀ MET-min/week) in Block 3 produced a significant increment (*Δ*R^2^ = 0.012, *p* = 0.023), bringing total variance explained to 13.3% (adjusted R^2^ = 0.120).

**Table 3:** Hierarchical multiple linear regression for correlates of observed ISWT distance (*N* = 389).

Predictor	B (95% CI)	*β*	p
Block 1: Sex, age, BMI, nationality			
Male sex	232.3 (162.4 to 302.1)	0.323	<.001
Age, years	−4.4 (−17.2 to 8.3)	−0.034	0.495
BMI, kg/m^2^	−8.6 (−14.0 to −3.2)	−0.153	0.002
Kuwaiti nationality	−77.7 (−151.1 to −4.2)	−0.101	0.038
R^2^ = 0.116; adjusted R^2^ = 0.107; *Δ*R^2^ = 0.116; *Δ*F(4, 384) = 12.58; *p* < 0.001			
Block 2: + Depressive symptoms			
Male sex	223.2 (152.7 to 293.8)	0.310	<0.001
Age, years	−5.2 (−18.0 to 7.6)	−0.040	0.423
BMI, kg/m^2^	−8.5 (−13.9 to −3.1)	−0.152	0.002
Kuwaiti nationality	−76.4 (−149.7 to −3.1)	−0.100	0.041
PHQ-9 total score	−5.1 (−11.5 to 1.3)	−0.077	0.115
R^2^ = 0.122; adjusted R^2^ = 0.110; *Δ*R^2^ = 0.006; *Δ*F(1, 383) = 2.49; *p* = 0.115			
Block 3: + Physical activity — fully adjusted model			
Male sex	203.2 (130.9 to 275.5)	0.283	<0.001
Age, years	−5.2 (−18.0 to 7.5)	−0.040	0.420
BMI, kg/m^2^	−9.1 (−14.4 to −3.7)	−0.162	<0.001
Kuwaiti nationality	−76.3 (−149.2 to −3.3)	−0.099	0.040
PHQ-9 total score	−5.25 (−11.6 to 1.1)	−0.079	0.104
Log₁₀ IPAQ MET-min/week	32.4 (4.5 to 60.4)	0.113	0.023
R^2^ = 0.133; adjusted R^2^ = 0.120; *Δ*R^2^ = 0.012; *Δ*F(1, 382) = 5.20; *p* = 0.023; all VIF < 1.16			

B, unstandardised coefficient; CI, confidence interval; BMI, body mass index; IPAQ, International Physical Activity Questionnaire; PHQ-9, Patient Health Questionnaire-9; VIF, variance inflation factor; β, standardised regression coefficient. The primary outcome was observed ISWT distance (metres). Physical-activity volume was entered as log_₁₀_(total IPAQ MET-min/week + 1). Sex was coded male=1, female=0; Kuwaiti nationality coded 1 = Kuwaiti, 0 = non-Kuwaiti. Coefficients correspond to the sequentially fitted model shown in each block; the Block 3 panel presents the final fully adjusted model. Bold values indicate statistical significance (*p* < 0.05).

**Figure 2:**
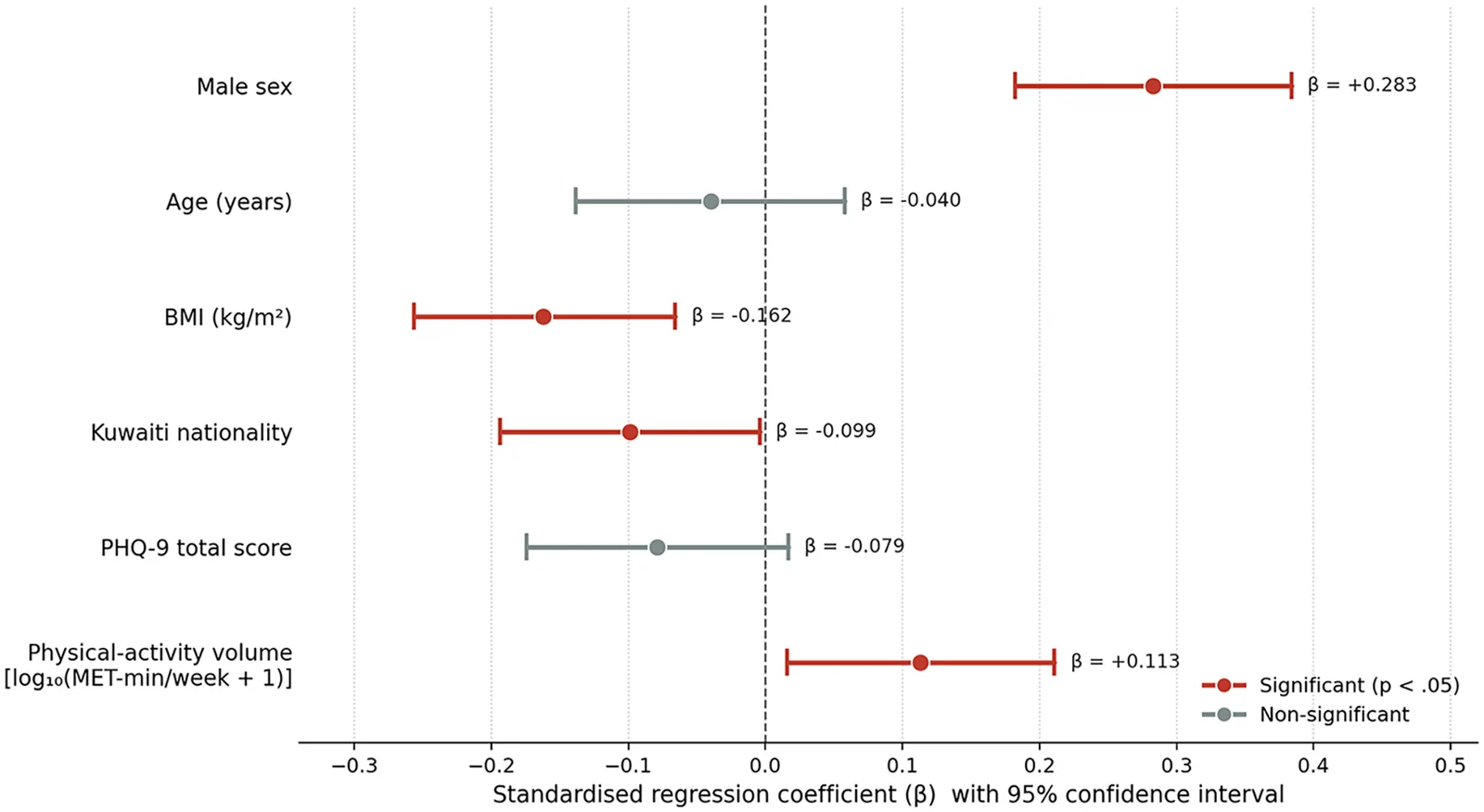
Adjusted correlates of observed ISWT distance in the fully adjusted primary hierarchical regression model (*N* = 389). Points are standardised regression coefficients (*β*); horizontal lines are 95% confidence intervals. Red markers denote statistically significant predictors (*p* < 0.05); grey markers denote non-significant predictors. Confidence intervals in this figure are displayed on the standardised scale for visual comparison. Exact unstandardised ordinary least-squares 95% confidence intervals are reported in Table 3, whereas BCa bootstrap confidence intervals are reported in the Results.

In the fully adjusted model (Figure 2), observed ISWT distance was independently associated with male sex [B = 203.2 m, 95% CI (130.9, 275.5), *β* = 0.283, *p* < 0.001], lower BMI [B = −9.1 m per kg/m^2^, 95% CI (−14.4, −3.7), *β* = −0.162, *p* < 0.001], Kuwaiti nationality [B = −76.3 m, 95% CI (−149.2, −3.3), *β* = −0.099, *p* = 0.040], and higher physical-activity volume [B = 32.4 m per log₁₀ unit, 95% CI (4.5, 60.4), *β* = 0.113, *p* = 0.023]. Depressive symptoms were not independently associated with observed ISWT distance [B = −5.25 m per PHQ-9 unit, 95% CI (−11.6, 1.1), *β* = −0.079, *p* = 0.104]. Age was not a significant predictor (*β* = −0.040, *p* = 0.420). Tolerance values ranged from 0.868 to 0.970 and VIFs from 1.031 to 1.152, indicating no meaningful multicollinearity. The modest explained variance indicates that substantial individual variation in observed ISWT distance was not captured by the measured correlates.

### 3.7 Robustness and exploratory analyses

BCa bootstrap replication (5,000 samples) of the fully adjusted model was consistent with the primary results: male sex [BCa 95% CI (115.2, 293.4), *p* < 0.001], BMI [BCa 95% CI (−14.1, −3.9), *p* < 0.001], Kuwaiti nationality [BCa 95% CI (−145.7, −6.8), *p* = 0.031], and physical-activity volume [BCa 95% CI (0.6, 64.5), *p* = 0.044] remained significant, whereas PHQ-9 did not [BCa 95% CI (−11.8, 1.2), *p* = 0.118]. In an age-restricted analysis (age 18–24 years, *n* = 378), the pattern of associations was unchanged, with physical-activity volume remaining significant (*p* = 0.019) and PHQ-9 non-significant (*p* = 0.136).

An exploratory model testing sex interactions indicated a significant sex×physical-activity interaction (*p* = 0.010; block *Δ*R^2^ = 0.024, *p* = 0.014), whereas sex×PHQ-9 (*p* = 0.139) and sex×BMI (*p* = 0.154) were not significant. Sex-stratified models (Supplementary Table 2) showed that physical-activity volume was independently associated with ISWT distance among females (*β* = 0.197, *p* < 0.001) but not among the smaller male subgroup (*β* = −0.110, *p* = 0.285); BMI was inversely associated with ISWT distance in both sexes (females *β* = −0.128, *p* = 0.026; males *β* = −0.242, *p* = 0.020). The overall stratified male model did not reach conventional statistical significance (*p* = 0.054) and its estimates should be interpreted cautiously.

Exploratory equation-derived analyses restricted to participants aged ≥21 years (*n* = 133; Supplementary Table 1) showed that physical-activity volume was consistently associated with equation-derived outcomes (percentage-predicted and observed-minus-predicted difference models, *p* = 0.005 and *p* = 0.007). PHQ-9 was not independently associated with any equation-derived outcome in this age-restricted subgroup (*p* = 0.305, 0.112, and 0.081, respectively). These findings should not be interpreted as establishing an independent association between depressive symptoms and observed ISWT performance.

## 4 Discussion

### 4.1 Principal findings

To our knowledge, this is the first study in the GCC region to apply an objective, field-based measure of functional exercise capacity among university students. Two principal findings emerge. First, observed ISWT distance was independently associated with male sex, lower BMI, higher physical-activity volume, and nationality, aligning with established correlates of fitness from large-scale systematic reviews (23). Physical-activity volume was the primary behavioural correlate examined and was independently associated with observed ISWT distance. Second, an exploratory sex×physical-activity interaction indicated that the positive association between activity and ISWT distance was evident among female participants but not the smaller male subgroup. Depressive symptoms were not independently associated with observed ISWT distance.

### 4.2 Functional exercise capacity and health context

Observed ISWT distance varied widely in this cohort. Comparisons with external healthy-adult reference values should be interpreted cautiously because available reference equations have not been validated in Kuwaiti university students. Low cardiorespiratory fitness is a strong, independent predictor of cardiovascular disease, type 2 diabetes, and all-cause mortality. An umbrella review of 26 systematic reviews representing over 20.9 million observations found that high CRF was associated with a 53% reduction in all-cause mortality risk (hazard ratio 0.47; 95% CI 0.39–0.56), with a dose-response relationship of 11%–17% reduction per 1-MET increment (1). The long-term significance of current fitness levels is further underscored by a meta-analysis of 55 prospective studies including 37,563 youths, which found that lower CRF in childhood and adolescence predicts higher BMI, waist circumference, insulin resistance, adverse lipid profiles, and overall cardiometabolic risk later in life (24). A 31-year cohort study further demonstrated that persistent physical inactivity from youth to adulthood is independently associated with clustering of cardiometabolic risk factors in adulthood (25).

Within the Kuwaiti context, these risks are compounded by structural and environmental factors. Physical inactivity prevalence exceeds 65% among Kuwaiti adults, NCDs account for approximately 72% of all deaths, with cardiovascular disease representing the single largest contributor (7). Overweight and obesity rates across GCC countries have nearly tripled over the past four decades, driven by rapid economic growth, urbanisation, and a lifestyle transition characterised by high-calorie diets and energy-saving technologies (26). Field-based assessment of functional exercise capacity is increasingly endorsed as a pragmatic tool for identifying at-risk youth in clinical and community settings (27).

### 4.3 Determinants of functional exercise capacity

The independent associations of male sex, lower BMI, and higher physical activity with greater absolute ISWT distance are consistent with the mechanistic and epidemiological literature. A systematic review of 78 observational studies found that male sex, lower BMI, lower body fat, and higher leisure-time physical activity were consistently and independently associated with higher CRF in adults (23). Sex differences in CRF are partly attributable to physiological factors including blood volume, haemoglobin concentration, and stroke volume (28): experimental evidence demonstrated that equalising blood volume and oxygen-carrying capacity between sexes eliminates approximately 19% of the observed gap in peak VO₂ (28). However, behaviour explains a substantial residual portion of the gap — in this study, 35.9% of males were in the high total-activity-volume group (≥3,000 MET-min/week) compared with only 7.7% of females (Table 1), consistent with documented structural and sociocultural barriers to female physical activity in Kuwait, including limited sex-specific facilities and cultural norms constraining public activity participation (29, 30).

Physical-activity volume was the primary behavioural correlate examined and was independently associated with observed ISWT distance (*β* = 0.113, *p* = 0.023; Table 3). This is consistent with evidence that even modest increases in activity produce clinically meaningful CRF gains, particularly in previously inactive individuals (27). The high prevalence of low study-defined total-activity volume in this cohort (52.7%) mirrors patterns across the MENA region, where a systematic overview found that only approximately half of adults meet recommended activity levels, with particularly low participation among females (31). Critically, physical activity may exert its greatest cardiometabolic benefit precisely in those with the lowest CRF: a systematic review in youth found that the association between physical activity and cardiometabolic risk was strongest among the least-fit individuals (32). In the present sample, the high prevalence of low study-defined total-activity volume supports the potential value of physical-activity promotion; however, this study does not establish population-specific CRF deficits. Depressive symptoms were not independently associated with observed ISWT distance in the fully adjusted model (*β* = −0.079, *p* = 0.104; Table 3), consistent with mixed findings in the literature on the fitness–depression relationship in healthy young adults (33, 34).

The inverse association between BMI and observed ISWT distance aligns with evidence that excess adiposity imposes both mechanical and metabolic constraints on exercise performance, including increased metabolic cost of movement, reduced exercise economy, and impaired VO₂max (35). Given that 49.3% of participants were overweight or obese (Table 1), the combined burden of excess adiposity and physical inactivity in this cohort creates a self-reinforcing cycle that compounds NCD risk.

### 4.4 Sex differences and the role of physical activity

Physical-activity volume was the primary behavioural correlate examined and was independently associated with observed ISWT distance. An exploratory interaction analysis indicated that this association differed by sex: the positive association between activity and ISWT distance was evident among female participants (*β* = 0.197, *p* < 0.001) but not among the smaller male subgroup (*β* = −0.110, *p* = 0.285), in which the overall stratified model did not reach conventional statistical significance (*p* = 0.054). These interaction and stratified analyses were not pre-specified, involve a substantially smaller male subgroup, and are reported cautiously in the Supplementary Material. A meta-analysis of 31 studies reported a moderate positive association between exercise motivation and cardiorespiratory fitness (r ≈ 0.24) among university students (36), and the marked sex difference in activity in this cohort—35.9% of males in the high total-activity-volume group vs. 7.7% of females (Table 1)—is consistent with documented structural and sociocultural barriers to female physical activity in Kuwait (29, 30).

Depressive symptoms were not independently associated with observed ISWT distance in the primary model (*β* = −0.079, *p* = 0.104), nor with any equation-derived outcome in the exploratory age-restricted supplementary analyses (all *p* > 0.08). PHQ-9 was retained as a pre-specified predictor and is reported transparently as non-significant; these findings should not be interpreted as establishing an independent association between depressive symptoms and observed ISWT performance. The observed associations among physical activity, fitness, and mood may reflect bidirectional relationships, shared determinants, or both; the cross-sectional design cannot establish temporal sequence or distinguish among these explanations.

### 4.5 Nationality differences

Kuwaiti nationality was independently associated with lower observed ISWT distance in the fully adjusted model (*β* = −0.099, *p* = 0.040), with Kuwaiti students walking shorter distances than non-Kuwaiti peers. This association may reflect unmeasured sociocultural, behavioural, environmental, or selection-related factors; the cross-sectional design cannot distinguish among these explanations. Al-Isa et al. (2011) identified reduced exposure to diverse lifestyle norms as associated with physical inactivity at Kuwait University, and highlighted limited sex-specific exercise facilities as a structural barrier (30). A theory-informed systematic review identified skills, social support, and cultural expectations as important modifying factors for physical activity among women in GCC countries (37), while qualitative research among Muslim university students in Qatar found that family health values, gender norms, and cultural expectations strongly shaped physical-activity engagement (38).

For descriptive context, Kuwait University reported that 31,771 of 44,098 registered students were female (72.0%) in academic year 2024/2025 (39), compared with 76.3% in the present sample—a modest over-representation of women of approximately 4.3 percentage points. Because the institutional figure includes all registered students across colleges and academic levels and does not account for voluntary participation or recruitment patterns, this comparison is descriptive and interpreted cautiously. Future research using mixed methods and multilevel modelling, and with attention to representativeness, is needed to clarify these mechanisms.

### 4.6 Strengths and limitations

A key strength is the use of the ISWT, a standardised, externally paced field test providing an objective and functionally meaningful measure of functional exercise capacity without requiring laboratory equipment, in contrast to the self-reported or non-exercise estimated fitness measures used in many comparable studies (27). The hierarchical modelling strategy and the comprehensive inclusion of sociodemographic, anthropometric, behavioural, and psychological variables enhance analytical depth. The data verification protocol confirmed that only one record required exclusion, and data integrity was maintained across all variables.

Several limitations must temper interpretation. First, the cross-sectional design precludes causal inference; the associations observed may reflect bidirectional or confounded relationships and temporal sequence cannot be established. Second, the ISWT is a field-based externally paced test that provides an indicator of functional exercise capacity rather than a direct measure of maximal cardiorespiratory fitness; observed ISWT distance is sensitive to motivational factors, test familiarity, and pacing ability (27); it may reflect physiological capacity together with these factors and should not be equated with direct measurement of maximal cardiorespiratory fitness or VO₂max. The combined clinical-symptom termination category (recorded as a single pre-existing form option) indicates that a mixture of physiological, symptomatic, and motivational factors may have influenced performance. Stop-reason labels were recorded as single assessor-selected categories. Time-synchronised nadir heart rate and SpO₂ values at the exact point of test cessation were not retained; therefore, stop-reason categories are reported descriptively and should not be interpreted as independently verified physiological events. Third, physical activity was assessed using the IPAQ-SF, which is known to overestimate activity levels and may introduce systematic measurement error (20); device-based accelerometry would more accurately characterise intensity and patterns. Fourth, the pragmatic convenience sample from a single university may limit generalisability; the sample was modestly more female (76.3%) than the Kuwait University registered-student population (72.0%; see Nationality differences), and findings should not be generalised to other GCC institutions. Fifth, key potential confounders including diet, sleep, screen time, and socioeconomic characteristics were not entered as primary exposures in the present analyses; these variables were collected and are reserved for subsequent papers in this programme of research. Finally, PHQ-9 scores reflect self-reported symptom burden rather than clinical diagnosis, and the mean PHQ-9 score was in the mild symptom range; findings should not be generalised to clinically depressed populations.

### 4.7 Implications for practice and research

The high prevalence of overweight or obesity and low study-defined physical-activity volume supports the rationale for early NCD-prevention initiatives in Kuwaiti higher-education settings. Multi-level interventions tailored to the GCC context appear warranted. Intervention evidence from the region indicates that structured walking programmes can significantly increase physical activity levels (40), and trial evidence supports structured aerobic exercise interventions for improving both CRF and depressive symptoms in university populations (41). At the institutional level, curriculum-integrated physical education, credit-bearing activity courses, and accessible facilities — particularly for female students — could leverage the structured university environment to normalise active behaviours and address the documented barriers for women (30, 37). Given the bidirectional and cross-sectional nature of the data, combined physical and psychological approaches should be evaluated in prospective and interventional research rather than assumed to be effective. Future longitudinal and interventional studies should clarify temporal sequence and test whether structured physical-activity programmes improve observed ISWT performance and depressive symptom burden in this population (24, 25, 42, 43).

## 5 Conclusion

Observed ISWT distance was independently associated with male sex, lower BMI, higher physical-activity volume, and nationality among students at Kuwait University, consistent with established determinants of fitness. Physical activity was the primary behavioural correlate examined, and an exploratory sex×physical-activity interaction suggested that this association was evident among female but not male participants. Depressive symptoms were not independently associated with observed ISWT performance. These findings provide an objective baseline profile of field-assessed functional exercise capacity and selected NCD-related health markers in an under-studied GCC population. They support the potential value of campus-based physical-activity promotion and field-based functional-capacity assessment in Kuwaiti higher-education settings, while requiring confirmation in larger, more representative, and longitudinal studies before policy-level conclusions are drawn.

## Data Availability

The datasets generated and analysed during the current study are available from the corresponding author on reasonable request, subject to approval by the Kuwait University Health Sciences Centre Ethics Committee in accordance with the conditions of ethical approval (Approval No. VDR/EC-2025-114). Requests to access the datasets should be directed to ahmad.salman@ku.edu.kw.
