# Supplementary material for: Field-assessed functional exercise capacity and selected NCD-related health markers among students at Kuwait University: a cross-sectional study using the incremental shuttle walk test
Source: Front Sports Act Living. 2026 Aug 12;8:1852703. doi: 10.3389/fspor.2026.1852703 (PMC13508158; doi:10.3389/fspor.2026.1852703)
Supplement: Supplementary file 1 [file Datasheet1.docx]

**Supplementary Material**

**Supplementary Table S1. Exploratory equation-derived analyses restricted to participants aged ≥21 years (n = 133).**

| **Predictor** | **(a) Adjusted for predicted B (β; p)** | **(b) Difference model B (β; p)** | **(c) % predicted B (β; p)** |
| --- | --- | --- | --- |
| PHQ-9 total score | −4.73 (−.069; .305) | −7.75 (−.135; .112) | −1.07 (−.148; .081) |
| Physical activity (log₁₀ MET-min/week) | 45.44 (.153; .024) | 57.93 (.234; .007) | 7.53 (.241; .005) |

*Equation-derived outcomes were calculated using the Azman et al. (2023) reference equation for healthy Asian adults aged 21–80 years (age-restricted subgroup, n = 133). Each cell shows the unstandardised coefficient B, the standardised coefficient β, and the p value. Three exploratory models are shown: (a) observed ISWT distance adjusted for predicted distance; (b) observed-minus-predicted difference; and (c) percentage of predicted ISWT distance. Each model was additionally adjusted for Kuwaiti nationality. Physical-activity volume (log₁₀ MET-min/week) was consistently and significantly associated with equation-derived outcomes across all three models. Depressive symptoms (PHQ-9) were not independently associated with any equation-derived outcome. All three models simultaneously included PHQ-9 total score, log₁₀-transformed IPAQ MET-min/week, and Kuwaiti nationality; model (a) additionally included predicted ISWT distance. These analyses are exploratory and are not applied in the main analysis because the reference equation was not validated in this population or in participants younger than 21 years and incorporates an effort-dependent ΔHR term. Negative values of the observed-minus-predicted difference indicate observed ISWT distance below the equation-derived predicted distance.*

**Supplementary Table S2. Sex-stratified multiple linear regression for observed ISWT distance.**

| **Predictor** | **Female β** | **Female p** | **Male β** | **Male p** |
| --- | --- | --- | --- | --- |
| BMI (kg/m²) | −.128 | .026 | −.242 | .020 |
| PHQ-9 total score | −.066 | .254 | −.148 | .161 |
| Physical activity (log₁₀ MET-min/week) | .197 | <.001 | −.110 | .285 |

*Standardised coefficients (β) from sex-stratified models (female n = 297; male n = 92). The female model was statistically significant overall (R² = .059, p = .003); the male model did not reach conventional statistical significance overall (R² = .117, p = .054) and its estimates should be interpreted cautiously given the smaller subgroup. Physical-activity volume was entered as log₁₀-transformed total MET-min/week. Age and Kuwaiti nationality were included in both models; coefficients for the variables most relevant to the sex comparison are shown.*

**Supplementary Table S3. Termination-reason frequencies (descriptive).**

| **Termination category** | **Overall n (%)** | **Female n** | **Male n** |
| --- | --- | --- | --- |
| Inability to maintain pace / missed two shuttles | 115 (29.6%) | 90 | 25 |
| Combined clinical symptoms | 115 (29.6%) | 74 | 41 |
| Fatigue or breathlessness | 75 (19.3%) | 59 | 16 |
| Assessor-recorded age-predicted maximum-heart-rate stop criterion | 63 (16.2%) | 59 | 4 |
| Assessor-recorded oxygen-saturation stop criterion | 14 (3.6%) | 13 | 1 |
| RPE 9–10 with inability to continue | 1 (0.3%) | 1 | 0 |
| Free-text “Other” entries (test completion) | 6 (1.5%) | 1 | 5 |
| Total | 389 (100%) | 297 | 92 |

*Termination status was recorded using six predefined form options and an optional free-text “Other” field. Frequencies are from the analytic dataset. Sex-specific counts are reported descriptively only; no inferential comparison is interpreted because of sparse category counts (for example, one participant in the RPE 9–10 category and six free-text “Other” entries). Clinical symptoms were recorded as one pre-existing combined response option (chest pain, dizziness, cramps, or severe dyspnoea) and could not be disaggregated retrospectively. Stop-reason labels were recorded as single assessor-selected categories; time-synchronised nadir heart rate and SpO₂ values at the point of test cessation were not retained, so categories are descriptive and should not be interpreted as independently verified physiological events.*

**Supplementary Figure S1.** Regression diagnostic plots for the fully adjusted primary model of observed ISWT distance (N = 389): (A) histogram of standardised residuals with a superimposed standard-normal curve; (B) normal probability (P–P) plot of standardised residuals; and (C) standardised residuals against predicted values. Visual inspection of the residual plots did not indicate major departures from normality or a systematic residual pattern. No case exceeded the absolute standardised-residual threshold of 3.0 in SPSS casewise screening. Plots depict the residual behaviour of the fitted model as generated in IBM SPSS Statistics.
